# Real-Time Detection and Feedback of Canonical Electroencephalogram Microstates: Validating a Neurofeedback System as a Function of Delay

**DOI:** 10.3389/fnsys.2022.786200

**Published:** 2022-02-25

**Authors:** Tomohisa Asai, Takamasa Hamamoto, Shiho Kashihara, Hiroshi Imamizu

**Affiliations:** ^1^Cognitive Mechanisms Laboratories, Advanced Telecommunications Research Institute International (ATR), Kyoto, Japan; ^2^Graduate School of Frontier Biosciences, Osaka University, Osaka, Japan; ^3^Department of Psychology, Graduate School of Humanities and Sociology, The University of Tokyo, Tokyo, Japan

**Keywords:** neurofeedback, delay, EEG microstates, control, sense of agency

## Abstract

Recent neurotechnology has developed various methods for neurofeedback (NF), in which participants observe their own neural activity to be regulated in an ideal direction. EEG-microstates (EEGms) are spatially featured states that can be regulated through NF training, given that they have recently been indicated as biomarkers for some disorders. The current study was conducted to develop an EEG-NF system for detecting “canonical 4 EEGms” in real time. There are four representative EEG states, regardless of the number of channels, preprocessing procedures, or participants. Accordingly, our 10 Hz NF system was implemented to detect them (msA, B, C, and D) and audio-visually inform participants of its detection. To validate the real-time effect of this system on participants’ performance, the NF was intentionally delayed for participants to prevent their cognitive control in learning. Our results suggest that the feedback effect was observed only under the no-delay condition. The number of Hits increased significantly from the baseline period and increased from the 1- or 20-s delay conditions. In addition, when the Hits were compared among the msABCD, each cognitive or perceptual function could be characterized, though the correspondence between each microstate and psychological ability might not be that simple. For example, msD should be generally task-positive and less affected by the inserted delay, whereas msC is more delay-sensitive. In this study, we developed and validated a new EEGms-NF system as a function of delay. Although the participants were naive to the inserted delay, the real-time NF successfully increased their Hit performance, even within a single-day experiment, although target specificity remains unclear. Future research should examine long-term training effects using this NF system.

## Introduction

Recent neurotechnology has developed various methods for neurofeedback (NF), in which participants observe their own neural activity to be regulated in an ideal direction. This neuromodulation through long-term training could improve participants’ cognitive performance or even some clinical traits for people with mental disorders (see for review, Watanabe et al., 2017; Lubianiker et al., 2019). These previous findings suggest our mental adaptability, where the NF system serves to assist our innate ability to learn new mental states. Since neural activity itself does not produce any sensory feedback within the brain, the system can “externalize” that activity to be controlled by the participants themselves. This strategy has been technically developed in terms of brain-computer-interface (BCI), where participants can control the externalized “sensory feedback” of their neural activity through training.

Regarding the implementation of the NF system, there are two options: spatial or temporal priority over the neural representations of the brain. The former is achieved by functional magnetic resonance imaging (fMRI)-NF, while the latter is mainly achieved by electroencephalogram (EEG)-NF. In terms of clinical application, resting-state functional connectivity (FC) in fMRI analysis can be a target of fMRI-NF (i.e., FC-NF), since FC is assumed to be a biomarker for some mental disorders (e.g., Yamada et al., 2017). More recently, however, resting-state EEG signals have also been actively used in line this this, taking advantage of its high temporal resolution to complement the disadvantages of fMRI, which has a resolution of only a few seconds, making it difficult to provide immediate feedback. One useful EEG measure in the context of NF is the EEG microstate. EEG-microstates (EEGms) are spatially featured expressions that can be regulated through NF training (Diaz Hernandez et al., 2016) since recent studies have also suggested EEGms as biomarkers (e.g., da Cruz et al., 2020; Murphy et al., 2020).

**FIGURE 1:**
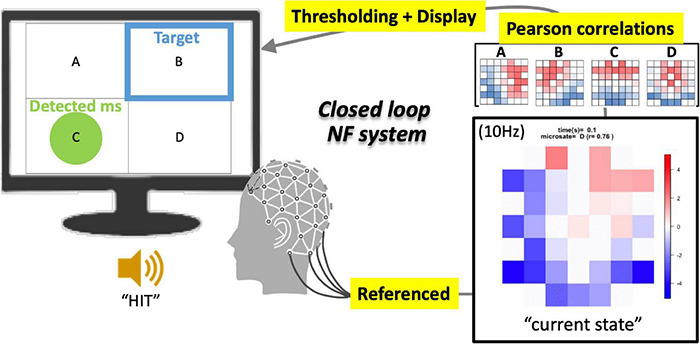
Schematics of EEGms neurofeedback system. The referenced bipolar signals are processed every 100 ms (10 Hz) into an epoch-averaged spatial pattern. This current state is compared with four EEGms templates on the basis of spatial similarity (Pearson’s correlation). When the largest value among the four absolute correlation coefficients is greater than 0.8 (for example) threshold, the display suggests a green circle in the corresponding area. The targeted state is suggested at the same time using a blue frame. If the green circle overlaps the blue frame, the participants receive a ringing sound as a reward.

**FIGURE 2:**
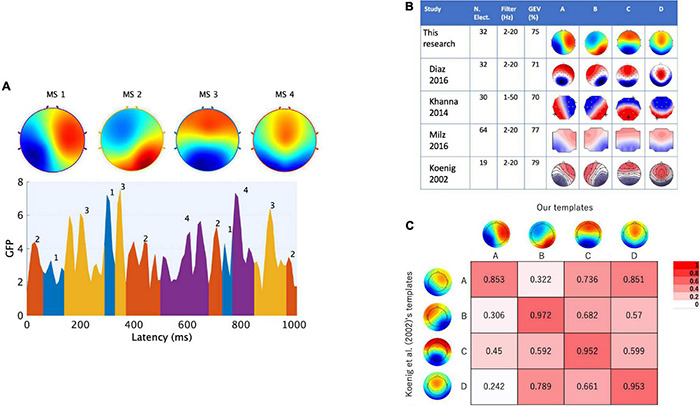
Canonical EEGms templates as the targeted states in neurofeedback. The microstate maps obtained from external participants under the eyes-closed resting state are shown. **(A)** Four maps are identified through a typical microstate analysis (upper) based on the GFP (global field power) peak dataset (lower). The sequences of microstate classes were determined by back-fitting to the data with the highest topographical correlation (see text for details). **(B)** These canonical templates were spatially congruent with previous studies, regardless of the EEG measurement or analysis tools. **(C)** Polarity-ignored spatial similarity (Pearson’s | r|) with normative templates in which both configurations were interpolated onto 67 × 67 grids.

Accordingly, the current study was conducted to develop an EEG-NF system for detecting “canonical EEGms” in real time (see Figure 1 for an overview). Once simple resting-state EEG data are collected from a sufficient participant population, group-level common EEG states can be obtained. Previous studies have repeatedly reported that there are only four representative EEG states, regardless of the number of channels, preprocess procedures, or participants (Khanna et al., 2015). Although the optimal number of canonical states remains debatable (Seitzman et al., 2017; Michel and Koenig, 2018), the four most agreed-upon spatial patterns are often called msA, B, C, and D (Figure 2). Therefore, in this study, our 10 Hz NF system was implemented to detect all four msABCD and audio-visually inform participants of its detection, unlike a previous NF system for a specific microstate (Diaz Hernandez et al., 2016).

The EEGms are observed as quasi-stable potentials lasting 60–120 ms that represent whole-brain network activity (Michel and Koenig, 2018). The suggested procedure for EEGms analysis consists of two main stages: *clustering* and *labeling*. At the clustering stage, the standard deviation (global field power: GFP) among all electrodes is first calculated for each recording session. The time points of the greatest strength in the neuroelectric field, namely GFP peaks, are well-reasoned to best represent periods of momentary stability in the voltage topography (Zanesco, 2020). Consequently, it is preferable to select GFP peak points to achieve a high signal-to-noise ratio. Every spatial pattern at the local maximum of each GFP time series is accumulated as the GFP peak dataset for a participant group as a whole. An unsupervised learning algorithm, such as K-means, conducts clustering of the GFP peak dataset into optimal classes. Finally, group-wise common topographies are obtained as templates (i.e., centroid) for each class (typically, msA, B, C, and D). After that, the labeling procedure calculates the similarity between the templates obtained and every time point (including the GFP peak points) of the participants and labels one of them (e.g., msA) for all data points in a so-called “winner-take-all” manner (see section “Discussion”, Mishra et al., 2020). Since each topography should last for a while in a millisecond order, the original EEG data with multiple channels are now simply seen as a state transition pattern among four states, such as C, D, B, A, D, etc. As a result, the EEGms analysis typically reveals the frequency of the state (“occurrence”), the lasting of the state (“duration”), and transition patterns among the states (“transition probability”) for each recording session or each participant. These depicted features of EEGms can be compared among participant groups as potential biomarkers (de Bock et al., 2020; Perrottelli et al., 2021).

The current study examined the real-time effect of the developed NF system, which has been implemented to detect canonical msABCD (group-wise common topographies) and audio-visually inform participants of its detection. The audio-visual NF, however, was intentionally delayed for participants to prevent their control in learning (i.e., hit the targeted state; see section “Materials and Methods”). We hypothesized that an inserted delay would affect participants’ learning through the NF system due to their inability to utilize the feedback. Although EEG-NF is advantageous in terms of its temporal resolution in comparison with fMRI-NF, the real-time effect has not been examined well in the literature, especially for EEGms. Given that the EEGms might be assumed as basic components of consciousness, often referred to as “atoms of thoughts” (Koukkou and Lehmann, 1987; Lehmann, 1992; Lehmann et al., 1998; Changeux and Michel, 2006), and last for a short period of time, EEGms-NF should become less effective with a certain delay (e.g., over some hundreds of milliseconds). Aside from EEGms-NF, several studies have attempted to implement real-time EEG feedback (Zich et al., 2015; Pei et al., 2020). A recent study examined the effect of latency in visual NF that falls within the range of 300 to 1000 ms in terms of a parietal alpha rhythm (Belinskaya et al., 2020), and concluded that the delay is a crucial parameter that must be minimized to achieve the desired NF effect. This policy is also motivation to enhance the “sense of agency” for participants in their NF training: a feeling of “I am the origin of the sensory feedback” (Gallagher, 2000). In addition to many psychophysical studies that have demonstrated that the feedback delay clearly reduces both participants’ sense of agency (Asai and Tanno, 2007; Asai, 2016) and their performance in motor control tasks (Tanaka et al., 2011), Evans et al. also suggested that an inserted delay resulted in a reduced sense of agency over the externalized feedback through a motor imagery-based BCI system (Evans et al., 2015).

**FIGURE 3:**
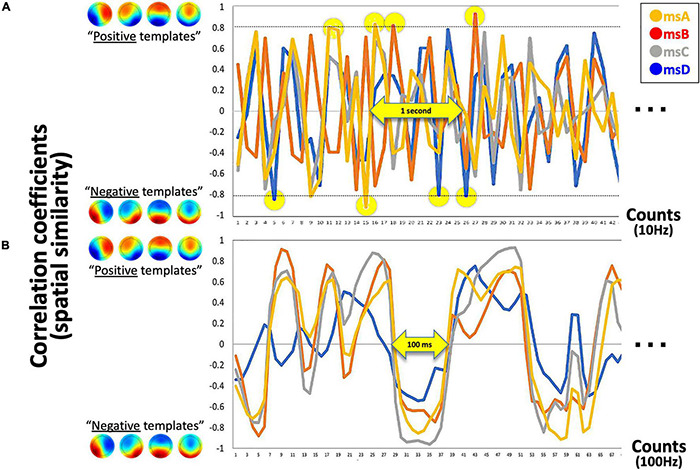
Detected canonical msABCD templates. **(A)** The exemplified time series of four correlation coefficients with template msABCD at 10 Hz is shown. The yellow circle indicates the detected canonical microstates in real-time (when r_*thr*_ = 0.8). **(B)** If the system is processed at 100 Hz (for comparison), a continuous state-transition dynamic can be observed. In this sense, our time-averaged 10-Hz system detects only spatio-temporally robust microstates.

**FIGURE 4:**
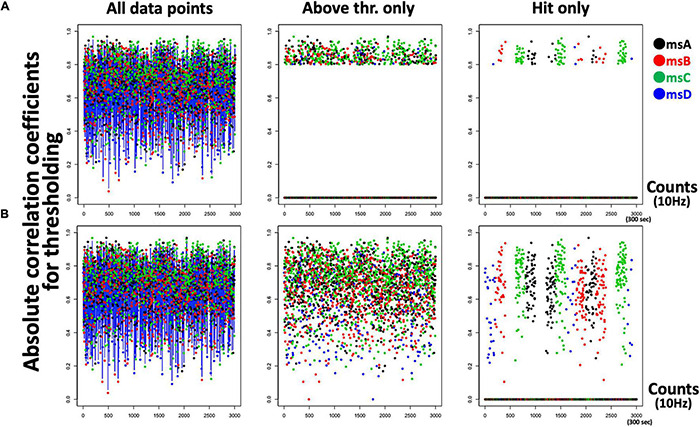
Outputs of the neurofeedback system. **(A)** A typical scatter plot of a 5-min session (left) showing visually informed (circle feedback) data points only (middle) and auditory-informed “Hits” only (right). **(B)** If the threshold is lowered (e.g., r_*thr*_ = 0.1 for comparison), participants receive too much feedback (visual for the middle panel and auditory for the right panel) to learn.

**FIGURE 5:**
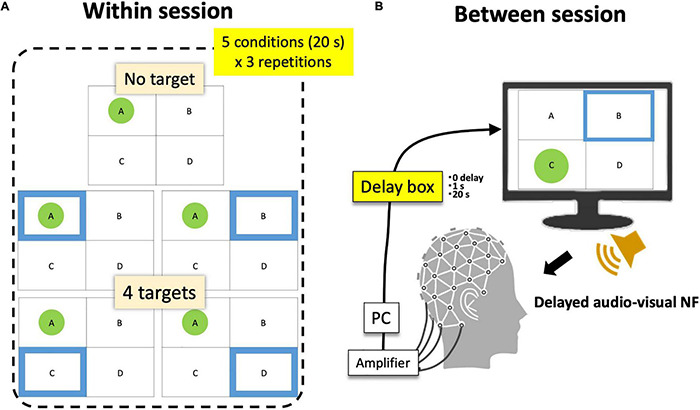
Within- and between-session conditions. **(A)** A session consists of five within-session target conditions × 3 repetitions. **(B)** Participants completed 6 sessions (3 between-session delay conditions × 2 repetitions). The order of conditions was randomized.

The aim of the current study was to develop a closed-loop NF system and validate it as a function of the inserted delay. Participants were instructed to attempt to make more “Hits” with audio-visual feedback (see Figures 3, 4 for the definition of “Hits”). However, in some conditions (i.e., “sessions” in the current case) the additional delay was inserted between participants’ neural activity and its externalized feedback in a secret manner (Figure 5 for experimental design). If real-time NF is effective for learning, participants’ Hit performance should be increased from the baseline period and from delayed conditions. In particular, given that each EEGms could be a different cognitive unit, as previously discussed, the controllability of each microstate in the real-time NF situation and robustness of the controllability to a feedback delay may be different depending on its cognitive responsibility. Therefore, we also compared the real-time effects of msA, B, C, and D in terms of each cognitive functionality.

## Materials and Methods

### Participants

A total of 18 young naive participants (9 females, mean age = 26.3) were recruited from the local community and paid for their participation. All participants reported normal or corrected-to-normal vision and hearing. All participants provided written informed consent before the experiments were conducted. The experiment was conducted in accordance with the principles of the Declaration of Helsinki. The protocol of the present study was approved by the local ethics committee (reference number is 20–144 for the Ethics Committee of ATR).

### Preparing for EEG-Microstates Templates

The templates used in our NF system to be matched with the participants’ current state were prepared in our previous study as a set of independent group-wise common topography, where 31 healthy people (15 females) in their 20 to 60 s were recorded under the four resting-like eye-closed conditions (resting state, meditated state, respiration counting, and heartbeat counting). Although the details for that experiment will be published elsewhere, the canonical four templates (msA, B, C, and D) were obtained from all those data, as briefly described below.

The EEG signals were recorded from 32 silver-silver chloride electrodes attached to a saltwater sponge-based electro-cap (R-net, Brain Products GmbH, Germany) and were placed at Fp1, Fp2, Fz, F3, F4, F7, F8, F9, F10, FC1, FC2, FC5, FC6, Cz, C3, C4, T7, T8, CP1, CP2, CP5, CP6, Pz, P3, P4, P7, P8, P9, P10, Oz, O1, and O2, according to the extended international 10–20 Systems. The reference and ground electrodes were placed at FCz and Fpz, respectively. We maintained impedances under 50 kΩ. EEG signals were amplified with a bandpass of 0.016–250 Hz and digitized at a 500 Hz sampling rate using an EEG recorder (BrainAmp ExG, Brain Products GmbH, Germany). The preprocessing of the EEG data was conducted in MATLAB (R2019b, MathWorks, United States) using the EEGLAB toolbox (EEGLAB2019_0; Delorme and Makeig, 2004). First, the raw signals were down-sampled to 100 Hz and filtered using a finite impulse response filter with a high pass of 2 Hz and a low-pass filter of 20 Hz. Then, we conducted a visual inspection to detect artifacts, including sweat, muscle, movement, and electrode trouble, to be removed manually. Channels with severe artifacts during the entire recording period were spatially interpolated. Furthermore, independent component analysis (ICA) was conducted to remove components with artifacts.

Microstate analysis was further applied to this clean dataset in MATLAB R2019b using the MST plugin for EEGLAB (Poulsen et al., 2018). We calculated the GFP and accumulated topographic voltage maps at local maxima (peaks) in the GFP time series (1000 GFP peaks per session as a default setting) for all participants to be analyzed by the modified k-means clustering algorithm, which ignores the polarity of the voltage maps. GFP peaks were used to generate initial maps for clustering to maximize the topographic signal-to-noise ratio. We defined the number of microstates as four, given that previous studies reported them as the most common (Khanna et al., 2014, 2015), although recent studies have also argued for the possibility of more canonical templates (e.g., Wackermann et al., 1993; Seitzman et al., 2017; Michel and Koenig, 2018).

### Developing EEG-Microstates-NF System

The EEGms-NF system was developed using OpenViBE (Renard et al., 2010) with embedded MATLAB code so that the same EEG cap and amplifier work with our templates (Figure 1). The input EEG signals (500 Hz) were first referenced and then bandpass-filtered (FIR, 2–20 Hz). After this minimum online denoising, our system implemented time-epoching, template-matching, thresholding, and displaying at 10 Hz. The epoching module in OpenViBE determines the time window that averages the online EEG signals as the “current” EEG topology that consists of a 32-dimensional vector, as a result. Since the typical duration of each microstate has been reported to be approximately 100 ms (Khanna et al., 2015; 60–120 ms, Koenig et al., 2002; Michel and Koenig, 2018), our 10 Hz system depicts a 100-ms–averaged topology for every process (i.e., for sequential 100-ms blocks without an overlap). This means that the only temporally stable microstate (duration of approximately 100 ms) should be detected for the following process. In this sense, a polarity reversal (e.g., A + to A−) should self-cancel the topography and should not be detected by the neurofeedback system at this stage.

Because our four prepared templates are also 32D vectors, the template matching was simply applied by calculating the spatial similarity (Pearson’s correlation in our case) between two 32D vectors for each template (i.e., the current vs. each template). As a result, the system output includes four time-series of correlation coefficients that range from −1.0 to + 1.0 in the definition. For the following, however, the absolute *r* value was used because the polarity is not of interest regarding EEGms (see above for definition of microstate). At this stage, we can define the threshold parameter for the absolute *r* values (see Figure 3A for our 10-Hz system). If the system is processed at 100 Hz (Figure 3B shows as an offline processing for comparison), we observe positive–negative fluctuation regardless of the template (Figure 3B) when we define positive-negative templates as anterior-posterior contrasts. Since our template A may not be optimal (see Figure 2C diagonals), spatial or temporal correlations between template A and B in the current case could be exceptional. For participants to learn to effectively “hit the target” by controlling their own neural state, audio-visual feedback should be appropriate in terms of its frequency (Figure 4). If the threshold is too low (e.g., r_thr_ = 0.1), participants receive feedback too often to learn (i.e., it is annoying, Figure 4B). Therefore, our pilot tests for the participants from our research group determined the threshold as approximately 0.8 and further individually adjusted (see below) in an explorative manner to reduce the frequency of feedback (Figure 4A). This also means that only the spatially robust microstate (topology near the templates) should be detected for the following process.

Finally, when the current topology passed the above-mentioned criteria for a spatio-temporally robust microstate, a display for participants indicated its detection (Figure 1) where participants always saw four frames with labels (A, B, C, or D). Only when that state was detected, a green circle appeared within the corresponding frame (the highest-r label is selected so that only one green circle was shown at a certain moment). At the same time, a blue frame was presented among four possible states as the target. When participants’ EEGms detected by the system hit the target (a green circle appears within a blue frame), a reward sound was played. Therefore, in short, participants were instructed to “generate more sounds” whereas the blue target randomly moved every 20 s. There were five target conditions (no target, A, B, C, and D) within a session of 5 min (20 s × 5 conditions × 3 repetitions) (Figure 5A).

### Behavior of the System Developed

In summary, the developed system was implemented to detect “four canonical microstates” in terms of the duration and spatial pattern, where those definitions (e.g., threshold) were parameterized. The current setting (time window = 100 ms and spatial similarity >0.8 ∼ 0.7) was intended to detect only spatio-temporally robust states, so that participants received roughly 1–3 instances of visual feedbacks per 1 s by default (mean ± SD: 2.7 ± 0.7 times/s) (Figure 4A) because EEGms transition should be an innate neural dynamic. Accordingly, only minimal online denoising was required, because potentially noisy states (e.g., eye blink, head motion, and other possible artifacts) must be ignored in the definition. We also confirmed that such intentionally added noise does not respond to our system regardless of msA, B, C, or D in preceding pilot tests. This indicates that participants could not use physical strategies; instead, they were encouraged to try only mental strategies to “make sounds”.

A previous study implemented the EEGms-NF system (Diaz Hernandez et al., 2016) in which the temporally weighted contribution of msD during the recent 1 s was fed back auditorily to participants. This policy was based on a typical microstate analysis, since each data point was assigned to msA, B, C, or D without polarity (often followed by temporal smoothing to ignore rapid transitions), whose templates were depicted individually. The current NF system, however, has a different approach to promote its real-time status on the basis of our simpler definition of the “state.” We need only a recent 100-ms data if the state lasts approximately that duration. This means, in turn, that a polarity reversal and a rapid mixture among msABCD should self-cancel the topography and should not be detected by the neurofeedback system. Therefore, our current system is conservative where only spatio (*r* > 0.8 approximately)-temporally (100 ms approximately) stable states should be detected at every 10 Hz epoching process. Other possibilities, including mistiming, polarity reversal, mixture among msABCD, and intrinsic/extrinsic noisy states, were ignored. In addition, our audio-visual feedback achieves supervised learning for participants who are aware of their current state independent of the target state (group-wise common topography). The difference between them can serve as a prediction error to be minimized through learning. Real-time feedback would contribute to the calculation of prediction errors with a valid temporal correspondence between the current state and the target. Therefore, we hypothesized that participants could learn to make more Hits through real-time feedback of the system, compared with delay-inserted conditions, as shown below.

### Inserting Delay for System Validation

For that purpose, we additionally inserted an audio-visual delaying hardware (SS-FBDL82, SPORTS SENSING Co., LTD., JAPAN) between the NF system and both the monitor and speaker (Figure 5B). The inserted delay parameters were 0, 1 s, or 20 s and were manipulated session-wise in a manner blinded to the participants. They repeated six sessions in total (three conditions × two repetitions in random order). Accordingly, participants had three delay conditions as a between-session factor and five target conditions as a within-session factor. Previous studies have suggested that approximately a 1-s delay or epoching may be long enough to disturb or reduce the effect of EEG-NF (Mulholland et al., 1979; Belinskaya et al., 2020). Furthermore, a 20-s delay was intended to make a total discrepancy between the current and target state since the target moves every 20 s (for example, participants see the target msA then try to make an msA state of their own, but the presented visual feedback was for the previous target condition other than msA). If the functionality of the targeted microstate was not perceptual, but more cognitive, a 1-s or even 20-s delay might be acceptable, but in that case (especially for a 20-s delay), this is not likely a pure EEG-NF effect, but should include a task general effect similar to the results of a cognitive workload (see section “Discussion”).

Participants first received instructions regarding EEG measurement and the necessity to remain immobile during experiments and about microstates in terms of controlling their occurrence. They were then instructed to make more Hits (a green circle within a blue frame) by changing their conscious states in an explorative manner for which success was also indicated by auditory ringing feedback. First, a 1-min practice session was conducted using the same system without additional delay, where the experimenters visually checked the response of the system (i.e., audio-visual feedback) to determine individual thresholds among 0.8, 0.75, and 0.7, to absorb potential individual differences. Because the system responsiveness could be affected by several factors [a cap condition with potentially bad channels or fit of the cap to the participant’s head (S, M, or L size) as well as individual differences in participants’ innate states in terms of the similarity with the canonical templates]. Six participants for each threshold were determined as a result. Since this was a simple manipulation to roughly equalize feedback frequency among participants, the effect of the threshold would not be examined (see also Discussion for further update of the procedure). After the practice session, the participants repeated six sessions with or without delay insertion, as mentioned above. Participants were also required to answer a question regarding subjective controllability of their own neural state toward each EEGms (A, B, C, or D) as well as their sleepiness using a 5-point Likert scale after each session. This was expected to be a proxy measure for evaluating participants’ sense of agency over the EEGms-NF. The total time for the current experiment per subject was 2 h.

## Results

The templates we obtained are shown in Figure 2, where the labels of maps were named according to previously established studies (i.e., typically msA, B, C and D; Lehmann et al., 1987; Koenig et al., 2002; Khanna et al., 2014, 2015; Diaz Hernandez et al., 2016; Milz et al., 2016; Michel and Koenig, 2018). The topography showing a left posterior to right anterior orientation was determined as msA, the right posterior-left anterior orientation was msB, the anterior-posterior orientation was msC, and the fronto-central extreme location was msD. The spatial configurations of these four maps were highly similar to those previously described and frequently cited in EEG microstate research (Koenig et al., 2002) (Figure 2B). Comparing each two maps with the corresponding label assignment (Brodbeck et al., 2012), we achieved high spatial correlation coefficients (0.85–0.97, Figure 2C). Furthermore, we calculated the global explained variance (GEV) of the templates. The GEV is one of the parameters that can be used to evaluate whether this set of maps is reasonable as a canonical template. It provides a metric of how well the selected template maps account for the variance of the entire dataset (Poulsen et al., 2018). The higher the GEV, the better the explanation of the entire dataset. As a result, our template of four cluster maps explained 75% of the variance. Referring to previous studies (Figure 2B), the GEV was 71% in Diaz Hernandez et al. (2016), who recorded and preprocessed EEG signals with the same number of electrodes and filter settings as ours, 70% in Khanna et al. (2014) (they used a wider bandpass filter than ours), and 77% in Milz et al. (2016) (they used a larger number of electrodes than ours). Therefore, we obtained canonical microstates that were congruent with previous observations. These templates were used in our NF system to be matched with participants’ EEG states in real time.

### Questionnaire and Raw Hit Scores

**FIGURE 6:**
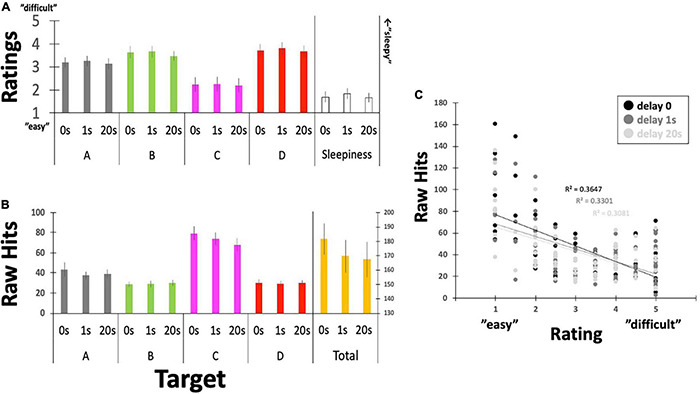
Questionnaire ratings for controllability and the raw number of Hits. **(A)** Participants reported their subjective feelings regarding “controllability” over the targeted EEGms and their sleepiness on the five-point Likert scale. **(B)** Participants’ raw Hits performance for each EEGms target. **(C)** A plot between the number of raw Hits and the Likert rating, including the delay conditions. Error bars indicate ± SE.

Participants’ raw reports about their subjective controllability over EEGms based on their Hit performance are summarized in Figure 6 and Table 1. The current results are congruent with some previous studies in terms of the statistical features of the four EEGms. For example, msC is reportedly the most dominant at default, especially in young, healthy participants (Tomescu et al., 2018). In line with this, the current participants’ raw number of Hits for msC was higher than that for msA, B, or D, regardless of the inserted delay (Figure 6B). The two-way ANOVA (4-target within conditions × 3-delay within condition) revealed significant main effects of the target and an interaction [*F*(3,51) = 24.2, *p* < 0.0001, and *F*(6,102) = 2.36, *p* = 0.0355], while the main effect of delay missed significance [*F*(2,34) = 24.2, *p* = 0.0501]. Multiple comparisons with Ryan’s method for the main effect of the target indicated that msC was significantly increased compared to msA, B, or D, respectively (ps < 0.05). Regarding the interaction, the simple main effect of delay on msC was significant [*F*(2,136) = 7.90, *p* = 0.0006]. The main effect of delay on the total Hits was significant [one-way ANOVA, *F*(2,34) = 3.36, *p* = 0.0466]. Accordingly, participants’ subjective feelings about the difficulty in controllability were also reduced for msC (Figure 6A). The two-way ANOVA (4-target within conditions × 3-delay within condition) revealed a significant main effect of target [*F*(3,51) = 7.93, *p* = 0.0002], while that of delay or the interactions were not significant [*F*(2,34) = 0.49, *p* = 0.6189, and *F*(6,102) = 0.09, *p* = 0.9969, respectively]. Multiple comparisons with Ryan’s method indicated that msC was significantly reduced from msA, B, or D (ps < 0.05). In addition, sleepiness reports did not differ among 3-delay conditions [one-way ANOVA, *F*(2,34) = 0.69, *p* = 0.5072].

**TABLE 1:** Descriptive statistics for participants’ raw Hits and ratings.

(A) Raw hits					(B) Ratings				
Descriptive statistics	Mean	Std. deviation	Minimum	Maximum	Descriptive statistics	Mean	Std. deviation	Minimum	Maximum
Total_0s	181.6	46.96	111	281	A_0s	3.194	0.957	1	5
Total_1s	169.6	49.74	93.5	268.5	B_0s	3.639	1.148	2	5
Total_20s	167.3	54.13	89.5	266	C_0s	2.222	1.396	1	5
msA_0s	43.39	31.25	17	160.5	D_0s	3.722	1.166	1.5	5
msA_1s	37.42	16.72	17	81	Sleep_0s	1.694	1.002	1	3.5
msA_20s	39.08	18.79	17	99.5	A_1s	3.25	1.018	1	5
msB_0s	28.81	11.68	15.5	61.5	B_1s	3.667	1.098	1.5	5
msB_1s	29	14.39	9	56.5	C_1s	2.25	1.364	1	5
msB_20s	30.17	12.65	9.5	56	D_1s	3.806	1.202	1.5	5
msC_0s	79.17	30.64	39	149	Sleep_1s	1.833	1.043	1	4
msC_1s	73.89	27.68	42.5	127.5	A_20s	3.139	1.026	1	5
msC_20s	67.89	29.08	37.5	136	B_20s	3.472	0.899	2	5
msD_0s	29.97	15.29	5	64	C_20s	2.194	1.33	1	5
msD_1s	29.14	13.87	2.5	54.5	D_20s	3.667	1.125	1.5	5
msD_20s	30	12.56	8	60.5	Sleep_20s	1.667	0.874	1	4

This outcome-dependent relationship between NF or BCI performance and perceived agency has been examined previously (Evans et al., 2015; Caspar et al., 2021). In particular, when the desired outcome is achieved, participants feel agency even if that outcome is not achieved by their own neural activity (Evans et al., 2015). Similarly, in the current study, it seems that agency ratings might have simply been reflected on the actual number of Hits by receiving or counting the sounds (e.g., for msC). Figure 6C clearly depicts the negative correlation between them with collapsing four target conditions, regardless of the inserted delay. However, regarding the effect of delay, there was no difference in ratings for either controllability or sleepiness, although Hit performance could be modulated by the inserted delay (see below for details). This suggests, in turn, that participants were not aware of the inserted delay, unlike many behavioral studies, when even short-time delays are easily detectable (Asai and Tanno, 2007). In such a situation, participants’ perceived agency is correlated with the detection of delay, which makes it difficult to eliminate the possibility that the reduced agency also affects participants’ motivation to learn. In this sense, no difference in their rating for the inserted delay (as a manipulation check) should be an important controlling result, especially in the current study.

### The Relative Hits as a Function of Delay

**FIGURE 7:**
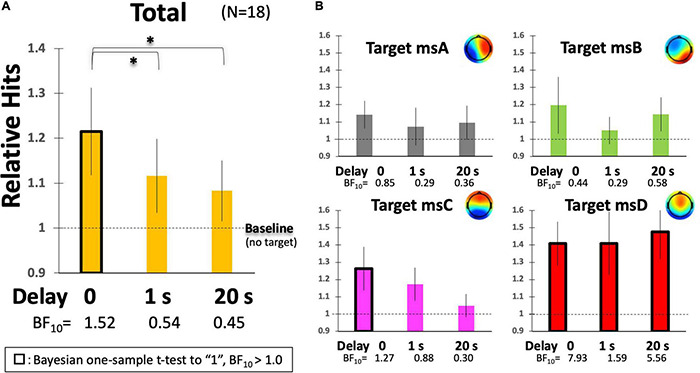
Participants’ Hit performance as a function of delay. **(A)** The relative magnification of total Hits in comparison to the individual baseline (no-target condition). **(B)** The same indices are calculated, respectively, for msA, B, C, and D. Error bars indicate ± 1 SE. *p < 0.05 in ANOVA.

To further examine the effect of delay on participants’ Hit performance, the raw scores were individually standardized based on the baseline period (no-target condition, see Figure 5A) because raw Hits by default have individual differences and also depend on the system threshold (r_thr_ = 0.8, 0.75, or 0.7) that were not strictly determined individually in the current study. Figure 7 indicates the relative Hits score, the relative magnification in comparison to the individual no-target condition without delay, as a function of the inserted delay. Our results clearly suggest that the real-time effect was observed only under the no-delay condition in the total score (Figure 7A). The relative Hits significantly increased from the 1- or 20-s delay conditions. A one-way ANOVA revealed that the main effect of delay was significant [*F*(2,34) = 4.86, *p* = 0.0139] with a difference between delay-0 and -1, and delay-0 and -20 revealed by a *post hoc* multiple comparisons using Ryan’s method (ps < 0.05). Furthermore, the two-way ANOVA (4-target within conditions × 3-delay within condition) revealed that the main effect of the target was significant [*F*(3,51) = 3.94, *p* = 0.0128], while that of delay and interactions were not [*F*(2,34) = 1.05, *p* = 0.3601, and *F*(6,102) = 1.04, *p* = 0.4032, respectively]. The relative Hit for msD was significantly increased from msA, B, and D, regardless of the delay, according to a multiple comparisons using Ryan’s method (ps < 0.05), suggesting a potential difference in the functionality of msABCD in terms of the responsiveness of our NF system with the inserted delay (Figure 7B).

For the individual EEGms, the increase from the baseline was the significant contrast of interest. For that purpose, we applied a Bayesian one-sample *t*-test to the baseline to avoid repetition of multiple tests (e.g., multiple Student’s *t*-tests). When we took a two-sided alternative hypothesis (H1:δ ≠ 1, since the relative Hits could be lower than 1) against the null hypothesis (H0:δ = 1), a Bayesian one-sample *t*-test with JASP (JASP Team, 2019) revealed weak but interpretable evidence (BF_10_ > 1) under the no-delay condition for the total, msC, and msD (as well as under the delay-1 or -20 condition for msD). For example, BF_10_ = 1.52 for total score without delay, indicates that the data are approximately 1.5 times more likely to occur under H1 than under H0, suggesting at least weak evidence in favor of H1 (a Bayes factor between 1 and 3 is considered weak evidence, but this range might be common in behavioral sciences). A confirmatory non-parametric *t*-test with Wilcoxon signed-rank suggested the same results, where the five conditions with black-framed bars in Figure 7 were significantly different from the baseline (ps < 0.05). Accordingly, when the Hits were compared among the msABCD, each cognitive function could be characterized. The total result is congruent solely with the msC, in which only the no-delay condition increased significantly above the baseline. Although a similar trend was detected for msA and msB, the difference was not significant. The msD is especially interesting, given that even a 20-s delay elevated the relative Hit performance compared with baseline (no-target specified), suggesting its general task-positive functionality. This in turn indicates the necessity of using a task-general baseline (see below); a previous study suggested the importance of using multiple baselines for assessing a training effect (Alkoby et al., 2018).

### Target Specificities for Each Delay Condition

**FIGURE 8:**
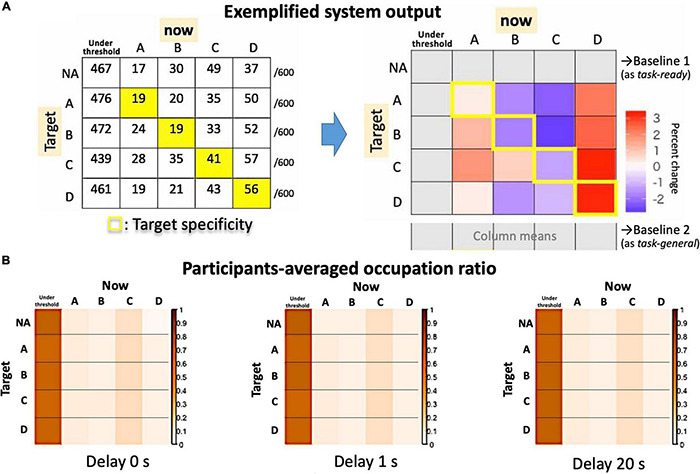
System outputs as an asymmetrical matrix. **(A)** The raw counts for an exemplified session (left) and the summarized matrix for the target specificity are shown in contrast to possible baselines (right). **(B)** Actual participants’ averages as occupation ratios for each delay condition.

The output of the system is summarized in Figure 8, in which a 4 × 4 (originally 5 × 5) matrix compares the Hits (diagonal components) and Misses (non-diagonals). For the group average, we assume that the “no-target” condition serves as a “task-ready” state rather than a resting state (Figure 8B), since the occupation ratio is almost that same as other “target” conditions regardless of the delay. Indeed, participants could observe their own EEG states as visual feedback even during the no-target period. Therefore, the individual baseline is needed to examine a small effect from real-time feedback on learning (i.e., the effect of a delay) within a single-day experiment. Since the definition of the baseline might change the results, the “task-general” baseline was also used for comparison with target conditions (Figure 8A, right).

**FIGURE 9:**
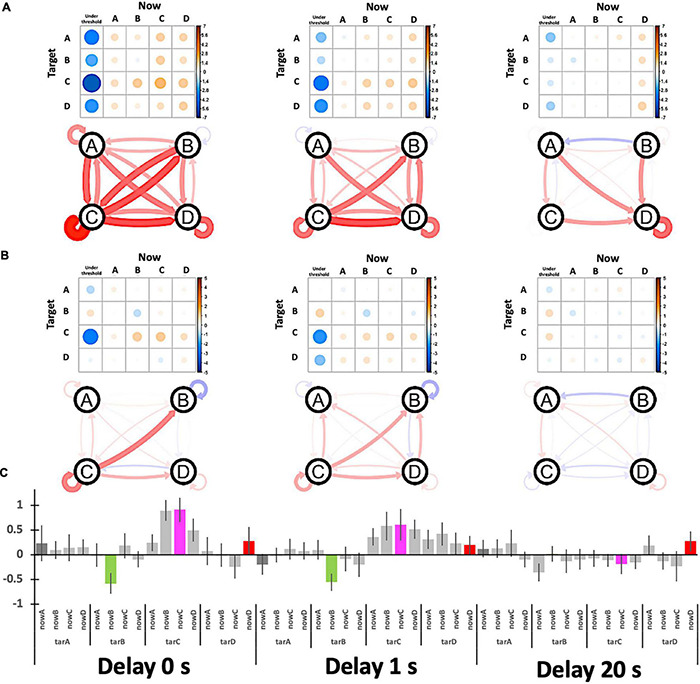
Participants’ relative performance for target specificity. **(A)** The baseline is the no-target period (“task-ready”) under the no-delay condition. **(B)** The baseline is the session average (the column-means for “task-general”) for each delay condition. The graphs show the relative Hits (self-recursive arrows for target specificity) and Misses (other arrows) where the nodes (A, B, C, and D) indicate the target. **(C)** The bar plot summarizes **(B)** with ± 1 S.E. error bars.

Figure 8 shows the target specificity analyzed with two possible baselines: the no-target period (“task-ready”) under the no-delay condition (similar to Figure 7) and the session average (the column means for “task-general”) for each delay condition (see Figure 8A, right). According to Figures 9A,B, the values are generally reduced with a delay regardless of being a Hit or Miss. A Hit for msC is the most sensitive to a delay, while msA exhibits the same tendency as msC in Figure 9A but msB shows the opposite in Figure 9B. The increase in msD evident in Figure 9A almost disappears in Figure 9B. A three-way ANOVA (4 targets × 4 currents × 3 delay conditions) applied to the “task-general” baseline (Figure 9C) shows that the effects of the delay and the target were significant [*F*(2,34) = 10.19, *p* = 0.0003; *F*(3,51) = 5.501, *p* = 0.0024, respectively]; the interaction between them was also significant [*F*(6,102) = 2.643, *p* = 0.02]. Accordingly, the target specificity (i.e., the three-way interaction or an interaction between the target and the detected state) was not specifically confirmed, although the main effect of a delay indicates a real-time effect from feedback, which was our initial purpose.

Although the target specificity is still unclear, that effect can be predicted to be learnable by participants through long-term training using this feedback system in a future study. This is related to their functional responsibility. Previous studies have suggested that the functionality of msA and msB are perceptual (auditory for msA and visual for msB); therefore, they might be passive and less controllable (oriented for sensory inflow, which is mainly audio–visual for humans), while msC and msD are cognitive (saliency or default mode for msC and attentive or executive for msD); therefore, they are active and more controllable (oriented for motor outflow with switching from internal to external awareness); below we discuss this functional difference in terms of delay sensitivity in our EEGms-NF system.

## Discussion

In the current study, we developed a new EEGms-NF system and validated it by examining the decline in participants’ performance as a function of delay. Although participants were presumably totally naive about the inserted delay as the result of the agency questionnaire also implies (Figure 6A), the real-time NF successfully increased their Hit performance in total (Figure 7A), even within a single-day experiment. This suggests that by using our NF system, participants could implicitly learn through a supervised manner how to control their own neural state, spatially represented in EEG channels, immediately during each no-delay session. Contrastingly, participants’ subjective agency was not modulated (i.e., reduced) even by a 20-s–delay, unlike many behavioral studies, given that they should not have been aware of the inserted delay. This indicates that we do not have an internal model of our own neural state (EEG state in our case) by default. Without this type of externalizing BCI system, it is difficult for us to estimate our own neural state or to compare the actual and desired states in forward modeling. This should be an essential factor why we do not feel a sense of control over BCI interactions in real time, where only outcome-dependent agency may be elicited (Evans et al., 2015). However, the current study further indicates that the use of an NF system that implements real-time sensory feedback like ours in long-term training for some months (such as daily motor behaviors), for example, an internal model over that system with subjective online agency can be developed. If the NF system represents our neural dynamics in nature, controlling or internalizing the system would mean that we could regulate our own neural state through the developed internal model (possibly even without the NF system). The EEGms, in this sense, are candidates for representing natural dynamics in the brain. Therefore, developing a BCI-based NF system in terms of online agency (or internal model) should be an important framework for future studies. For that purpose, our results suggest that minimizing feedback delays could be a worthwhile implementation for improving task learning (Belinskaya et al., 2020) such as motor learning (Tanaka et al., 2011).

In comparison with the preceding EEGms-NF system (Diaz Hernandez et al., 2016), there are several points to be discussed here. First, the templates in the system as the desired target state are group-wise topographies in our case, while in the previous study they were individually defined. This difference could be expected to result in different training outcomes. In our case, the spatial pattern itself was the target for participants’ learning, given that the individual EEGms must be slightly different from the group-wise EEGms (the “canonical” EEGms). We may observe the changes for individual EEGms moving toward group-wise EEGms through long-term training in future studies. However, individually defined templates should be easier for participants to induce, because they exhibit these patterns by default in nature, although the canonical EEGms are reportedly common across participants. Another difference in the implementation is sensory feedback. For supervised learning, participants were visually aware of their current and targeted state simultaneously. In addition to the visual feedback used in the previous study, the auditory feedback was provided to indicate Hits (successes in minimizing a prediction error) and to reward participants, as well. This audio-visual BCI monitors and externalizes whole-brain mass neural activity as EEGms to be regulated (Diaz Hernandez et al., 2016). This implementation enables us to treat four targets simultaneously (see Figure 1). In addition, the optimal frequency of NF is an important factor for participants’ learning so that the definition of EEGms has been further parameterized in terms of its duration and topographical robustness. Therefore, the difference to note is our definition of the state. Our system detects only spatio-temporally robust EEGms (approximately 100 ms for duration and r_*thr*_ = 0.8 for spatial similarity) so that strict online denoising was not necessary, which is always a nuisance in the “winner-take-all” definition of EEGms. Both noisy states and spatio-temporally less-robust states are ignored in our system. This largely helped the real-time implementation. However, if a polarity reversal or rapid transition is observed during the 100 ms (e.g., msA followed by msC and msD), the system ignores that state (i.e., as a mixture of A, C, and D, see Figure 3B). This is contrasted to a conventional offline EEG microstate analysis that has been implemented to handle this with polarity-ignored labeling (msABCD) and final temporal smoothing among labels (Poulsen et al., 2018). Recently, this classical approach of microstate labeling (“winner-take-all”) has been controversial (e.g., Shaw et al., 2019; Mishra et al., 2020). Some previous studies have intentionally introduced “unlabeled” states for this reason (e.g., Zanesco et al., 2021). In this sense, the current system was not developed to be matched with the classical labels, and there should be non-small differences between them depending on the analysis parameters, especially temporal smoothing of labels. Accordingly, our conservative system was not designed to detect every robust state but to not falsely detect them. We can imagine that 1-ms or 10-ms–averaged state is less reliable for learning and needs further temporal smoothing during a certain period. In this sense, we may update to a 100-Hz system using overlapping 100-ms blocks in a future study.

Regarding the potential differences among the four EEGms in responsible cognitive functionality, our results suggest some results congruent with previous studies as well as some new findings, especially in terms of neural controllability. First, for msD, the most frequently examined among the four EEGms, was reportedly increased through NF training for 10 days (Diaz Hernandez et al., 2016). Although that study was the first attempt to modify EEGms, msD is a specific target for future clinical application, since msD may be a biomarker for schizophrenia. Therefore, a link between schizophrenic symptomatology and the function of msD has often been discussed. Previous studies indicate that msD is functionally related to attentional control or central executive function that the frontoparietal network underpins. Simultaneous EEG-fMRI recordings suggest a link where the fluctuation of msD (time-series of spatial correlation to msD template) explains BOLD signals in the right-lateralized dorsal and ventral areas of the frontoparietal cortex (Britz et al., 2010). More recently, a simple mental arithmetic calculation increased participants’ duration of msD (Seitzman et al., 2017; Bréchet et al., 2019). If msD is responsible for this attentive cognitive load in general, our results are consistent with this. Even during 20-s–delay NF sessions (participants were not aware of this), participants tried to make more Hits so that the msD was increased compared to baseline. However, msC has often been discussed in contrast to msD as some previous studies indicate that the increased msD coincides with the decreased msC (e.g., Seitzman et al., 2017; Bréchet et al., 2019). Indeed, simultaneous recordings suggested that msC is neurally related to the default mode or saliency network, which is the functionally opposite network to the frontoparietal network (Britz et al., 2010; as a review, Seitzman et al., 2017; Michel and Koenig, 2018). In the current study, msC was the most delay-sensitive and controllable in real time. In this sense, saliency-related cognitive function may be a candidate for msC.

In contrast to the msC-D axis for controllable cognitive states, msA and B have been discussed as more perceptual functionalities. Simultaneous recordings suggest that msA is neurally related to the auditory region, while msB is related to the visual region (Britz et al., 2010). Recent studies also indicate msA for auditory perception as well as msB for visual perception, depending on the stimuli of modality or perceptual experiences (e.g., Cai et al., 2018; Bréchet et al., 2019; D’Croz-Baron et al., 2021). On the other hand, our results suggest that msA or B did not increase, even with real-time NF. These results indicate that the functionality of msA and B (or msA-B axis) is more perceptual; therefore, it might be passive and difficult to control intentionally. Although a more comprehensive understanding of four canonical EEGms is needed, since previous findings were given in a relatively sporadic matter, our results discriminated between msC-D and msA-B in terms of real-time controllability with NF. Our experiment did not examine each functionality directly and we do not assume that cognitive functionality has a 1:1 correspondence to each microstate, rather, microstate “dynamics” could be responsible to our cognition and perception (Michel and Koenig, 2018; Ruggeri et al., 2019, 2020). However, the result is interpretable in line with or extending the existing understanding of EEGms where the functionality of msA and B are perceptual (auditory for msA and visual for msB), and therefore, may be less controllable, while msC and D are cognitive (saliency or default mode for msC and attentive or executive for msD), and therefore, more controllable.

This study has some limitations for future applications. The current study was conducted within a single day for each participant. Accordingly, the effects of long-term training using the system are unknown, especially for the target specificity. Even within a session, the total Hit performance (and msC and msD) increased above the “task-ready” baseline (Figure 9A), suggesting that long-term training is predicted. If msC or msD is a candidate biomarker for some diseases (for example, schizophrenia), our system is clinically applicable for future studies as it is (without the inserted delay, of course). Although msA and msB appear more difficult to control according to the current results, they are still useful for examining the effect of long-term training, since a non-significant tendency for increasing was observed (Figures 7, 9). Another possible future approach would be to manipulate a parameter of threshold (spatial correlation r) individually, as well as msABCD, respectively. The former serves to absorb individual differences in an EEG cap condition, head fitness with a cap, and innate similarity with canonical templates. The latter serves to equalize the feedback frequency among msABCD because msC might occur more often innately. Examining thresholds could improve the balance between discriminability and detectability among msA, B, C, and D (Figures 3A,B). For this purpose, pre-training recording is required to determine the optimal thresholds and objective procedure. Whether this parameter should be changed daily through long-term training should also be considered. Even in this case, our system has already parameterized the threshold value. This should prove helpful, especially for specific participants with disorders who might have a reduced msD, for example, the system with a regular parameter should be less responsive to them than to healthy participants. Finally, the definition of a state as a canonical EEGms and the number of microstates are controversial (Michel and Koenig, 2018; Tarailis et al., 2021). Further studies should address this issue, since the current study used traditional definitions. Because our system was developed to make the template easily replaceable, new state definitions can be readily applied for participant learning (e.g., a 7-EEGms model).

In conclusion, the aim of the current study was to develop a closed-loop NF system and validate it as a function of the inserted delay. If real-time NF is effective for learning, the participants’ Hit performance should increase above baseline and from delayed conditions. Our results suggest that this is the case, although the target specificity is still unclear. Future studies can examine long-term training with this NF system, even for a specific population.

## Data Availability Statement

The raw data supporting the conclusions of this article will be made available by the authors, without undue reservation.

## Ethics Statement

The studies involving human participants were reviewed and approved by the Ethics Committee of ATR (reference number is 20–144). The patients/participants provided their written informed consent to participate in this study.

## Author Contributions

TA mainly designed the experiments, analyzed the data, and wrote the manuscript. TH and SK partially analyzed the data and wrote the manuscript. TA, TH, SK, and HI discussed the results and reviewed the manuscript. All authors approved the final version of the manuscript for submission.

## Conflict of Interest

The authors declare that the research was conducted in the absence of any commercial or financial relationships that could be construed as a potential conflict of interest.

## Publisher’s Note

All claims expressed in this article are solely those of the authors and do not necessarily represent those of their affiliated organizations, or those of the publisher, the editors and the reviewers. Any product that may be evaluated in this article, or claim that may be made by its manufacturer, is not guaranteed or endorsed by the publisher.
